# A short questionnaire for the assessment of quality of life in patients with chronic obstructive pulmonary disease: psychometric properties of VQ11

**DOI:** 10.1186/1477-7525-11-179

**Published:** 2013-10-25

**Authors:** Gregory Ninot, Franck Soyez, Christian Préfaut

**Affiliations:** 1 Laboratory Epsylon, EA4556 Dynamics of Human Abilities & Health Behaviors, University MONTPELLIER 1, Montpellier F-34000, France; 2 U1046 INSERM Physiologie et Physiopathologie du Coeur et du Muscle, University MONTPELLIER 1, CHRU Montpellier, Montpellier F-34295, France

**Keywords:** Chronic obstructive pulmonary disease, Brief questionnaire, Health-related quality of life, Validity, VQ11

## Abstract

**Background:**

There is a need for a validated short instrument that can be used in routine practice to quantify potential short-term change in Health-Related Quality of Life (HRQoL) in patients with chronic obstructive pulmonary disease (COPD). Our aim is to determine the validity and reliability of the VQ11 questionnaire dedicated to the routine assessment of HRQoL.

**Methods:**

181 COPD patients (40–85 yrs, I to IV GOLD stages) completed the VQ11, and several tests. One week later, 49 of these patients completed the VQ11 again.

**Results:**

Confirmatory factor analysis supported the two-level hierarchical structure of the VQ11 with 11 items covering three components and HRQoL at a higher level. The VQ11 showed good internal consistency and good reproducibility (r = 0.88). Concurrent validity showed significant correlations between VQ11 total scores and St George’s Respiratory Questionnaire-C (r = 0.70), Short Form-36 (r = -0.66 for the physical component and -0.63 for the mental component). We obtained significant correlations with MRC Dyspnea Grades (r = 0.59), the Hospital Anxiety and Depression Scale total score (r = 0.62), and the BODE index (r = 0.53).

**Conclusion:**

The VQ11 has good measurement properties and provides a valid and reliable measure of COPD-specific HRQoL. It is ready for use in routine practice.

**Clinical registration:**

The study was approved by the University of Montpellier 1 Ethics Committee and the Regional Ethics Committee (authorization number: A00332-53).

## Background

Self-administered questionnaires are required in order to estimate global outcomes of COPD [1]. Available disease-specific Health-Related Quality of Life (HRQoL) measures, mainly the St George’s Respiratory Questionnaire (SGRQ) [2], and the Chronic Respiratory Disease Questionnaire (CRDQ) [3], are reliable and valid, and widely used in clinical trials. There is increasing evidence that HRQoL questionnaires can also be useful in clinical settings [4]; however, existing instruments are lengthy and have complex scoring algorithms, making them poorly suited for routine use in clinical practice and repeated assessment. Most patients require short questionnaires (reducing fatigue, eliminating redundancy of items, and facilitating spontaneous responses).

A standardized, patient-centered assessment instrument covering key aspects of COPD HRQoL facilitates information gathering and improves communication between patient and clinician, particularly for general practitioners. An ideal instrument would identify specific areas of greater severity that would serve as focal points for targeted management or the evaluation of management goals, thereby improving both the process and outcome of care. The instrument must be sensitive enough to measure mild-moderate COPD [5], but also reliable [6,7], valid for evaluative studies, and useful for determining rehabilitation routines. Previous studies have shown significant changes in quality of life score between three and six months in patients with COPD participating in a rehabilitation program [8] or, after discharge from a rehabilitation program [9,10].

Recently, short self-administered questionnaires, the COPD Clinical Questionnaire (CCQ) [11], the Short Form Chronic Respiratory Disease Questionnaire (SF-CRQ) [12], the Visual Simplified Respiratory Questionnaire (VSRQ) [13], the COPD Assessment Test (CAT) [14], and the COPD specific HRQoL (VQ11) [15], have been validated with similar psychometric properties (Table 1) and some limitations.

**Table 1:** Psychometric properties of short HRQoL questionnaires for COPD patients

	**CCQ**[11]	**SF-CRDQ**[12]	**VSRQ**[13]	**CAT**[14]	**VQ11**[15]
Dimension	Symptom	Dyspnea	Total	Total	Total (HRQoL)
	Physical	Fatigue	(HRQoL)	(COPD impact)	Functional Psychological Social
	Mental	Emotion			
	Total	Mastery			
Items	10	8	8	8	11
Answer	Frequency	Frequency or intensity	Frequency or intensity	Frequency or intensity	Intensity
	Likert 6	Likert 6	Likert 11	Likert 6	Likert 5
Range	0 - 6 (Total)	1 - 14 per dimension	0 - 80	0 - 40	11 - 55
Item reduction	From 77 to 10	From 20 to 8	From 18 to 8	From 21 to 8	From 24 to 11
	Expert committee	Authors	Expert committee	Rasch	Confirmative factor analysis
Model	None	Principal component analysis	Principal component analysis	Rasch analysis	Structural equation modeling
α Chronbach	.91	.82	.84	.88	.83
Test-retest	.94	-	.77	.80	.72
r Total SGRQ	.71	-	-	.80	-
r FEV_1_	-.38	-.07 to -.28	-	-	-

In practice, the 8-item VSRQ and the 8-item CAT include no subscales. Conversely, the 8-item SF-CRDQ does not provide a total score. The SF-CRDQ, VSRQ and CAT mix answers for frequency and intensity, which could be difficult to distinguish in patients with COPD.

Conceptually, these instruments establish confusions to estimate the impact of COPD between health status and HRQoL. The concept of health status refers to the impact of health on the individual’s ability to perform daily life activities and to benefit from them [16]. HRQoL refers to the three broad dimensions of health: physical (e.g., autonomy, capacity, symptoms), psychological (e.g., pain, self-esteem, and symptoms), and social (e.g., social relationship, family relationship) [17]. Thus, an instrument dedicated to the measure of HRQoL needs to provide 3 dimensions (physical, psychological and social) and an overall total score.

Qualitatively, the SF-CRDQ and the CAT include information on daily symptoms, activity limitation and other physical manifestations of COPD (Table 2). These instruments are specific to the functional and psychological outcomes of COPD (symptoms, function and confidence in living at home), whereas the social effects produced by COPD can only be assessed by HRQoL measures [18]. The VSRQ and the CAT do not include any item on depression, which is prevalent in COPD patients and alters HRQoL [19]. The 10-item CCQ includes one social life question and relates directly to respiratory problems. The VQ11 is a short instrument that was designed to measure the functional, psychological and social aspects of COPD consequences and provide an overall score for specific HRQoL.

**Table 2:** Item coverage of short HRQoL questionnaires for COPD patients

**Dimension**	**Aspect**	**CCQ**	**SF-CRDQ**	**VSRQ**	**CAT**	**VQ11**
	Shortness of breath	2	1	1	1	1
	Fatigue		2	1	1	1
Functional	Activity limitations	3	1	1	1	1
	Phlegm	1			1	
	Cough	1			1	
	Chest tightness				1	
	Low self-confidence				1	1
Psychological	Anxiety	1	2	1		1
	Depression	1	1			1
	Sleep		1	1	1	1
	Sexual life trouble			1		1
Social	Life project limitation			1		1
	Social support lack					1
	Social life restriction	1		1		1

Psychometrically, for all the above instruments but the CAT and VQ11, the item reduction selection was made by a committee of experts and without the use of statistical analysis such as Confirmatory Factor Analysis. A previous study showed that the VQ11 has good content and internal properties [15]. This study was carried out to verify its construct’s validity and reliability in comparison to other short instruments (Table 1).

## Methods

### Participants

Participants were recruited from three pulmonary clinics and two medical offices between January 2008 and June 2009 using advertising flyers. To be included in the study, patients had to be aged between 40 and 85 years, and have an incompletely reversible limitation in airflow (forced expiratory volume in 1 s (FEV_1_) to forced vital capacity (FVC) ratio ≤ 70%) and I to IV GOLD stage (FEV_1_ < 80% th.). Patients with severe or uncontrolled comorbidities (unstable and/or uncontrolled cardiac disease, terminal disease, dementia, or an uncontrolled psychiatric illness) were excluded. 181 (123 males and 58 females) participated in the study after providing informed written consent. Demographic and clinical characteristics are listed in Table 3. The subjects did not participate in any other research study during this period. This study was approved by the University of Montpellier 1 Ethics Committee and the Regional Ethics Committee (authorization number: A00332-53).

**Table 3:** Clinical characteristics and HRQoL measures for 181 patients (123 males and 58 females)

	**Mean**	**SD**	**Min**	**Max**
*Sociodemographic and overall characteristics*				
Age (yrs)	61.4	9.8	37	85
BMI (kg/m^2^)	25.7	5.4	13.1	39.7
BODE score	3.5	2.4	0	10
Dyspnea MMRC	1.4	1.3	0	3
*Smoking history*				
Pack-years (smokers)	33.5	32.3	0.1	135.0
Pack-years (ex-smokers)	45.5	28.9	0.8	157.5
*Spirometry*				
Pre-BD FEV_1_ (ml)	1395	645	460	4090
FEV_1_ (% pred)	49.0	20.5	15	112
FEV_1_/FVC (%)	47.7	12.4	25	69
*Exercise tolerance*				
Dyspnea 6MWD end	6.0	2.1	2	10
6MWT distance (m)	470.9	122.1	90	812
6MWT distance (% pred)	70.9	17.3	14	121
*HRQoL Measures*				
SGRQ-C Symptoms score	54.3	18.9	6.8	97.3
SGRQ-C Activity score	55.3	22.7	7.3	100.0
SGRQ-C Impact score	34.2	18.7	4.2	89.1
SGRQ-C Total score	46.3	18.5	9.7	97.7
VQ11 Functional (3–15)	8.8	2.8	3	15
VQ11 Psychological (4–20)	10.2	3.2	4	18
VQ11 Social (4–20)	9.0	3.8	4	19
VQ11 Total score (11–55)	27.9	8.8	11	49
SF-36 Physical functioning	56.3	22.8	0	100
SF-36 Role physical	40.1	35.0	0	100
SF-36 Role emotional	55.7	40.2	0	100
SF-36 Energy/vitality	46.9	18.6	0	100
SF-36 Mental Health	64.0	15.3	20	100
SF-36 Social functioning	74.4	23.4	0	100
SF-36 Bodily pain	70.2	26.7	0	100
SF-36 General health perceptions	36.2	19.9	0	95
SF-36 Physical Component Scale	50.5	22.1	8.5	96.3
SF-36 Mental Component Scale	60.3	19.0	16.0	93.5
*Other Measures*				
HADS Anxiety score (0–21)	8.2	3.8	0	19
HADS Depression score (0–21)	6.0	3.4	0	17
HADS Total score (0–42)	14.2	6.3	1	33
Physical self-worth (1–6)	2.8	1.1	1.0	5.6

### Study design

Upon recruitment, the following examinations were performed for each participant: a clinical assessment, the collection of cardio-respiratory family history and number of exacerbations for respiratory (or other) reasons, a respiratory function examination (spirometry, blood gas analysis), an electrocardiogram, and a six-minute walk test (6MWT). Participants were also required to complete the experimental questionnaire, the external validity questionnaires (Medical Outcome Survey Short - SF-36, SGRQ-C, Hospitalization Anxiety Depression Scale - HADS, Physical Self-Worth - PSW) and a datasheet on their socio-cultural situation. Appointments were made with participants who were able and willing to undergo a follow-up assessment one week later. Forty-nine participants completed the VQ11 again to test its reproducibility. To ensure uniform assessments in this multicentric study (Montpellier, Paris, Osséja), the medical and scientific committee of the healthcare network developed recommendations and one teaching session for a standard protocol for instrument use and patient assessment.

### Completion of questionnaires

The participants completed the battery of self-administered questionnaires, presented in a randomized order, while resting between physical tests. Participants who took part in both sessions were examined by the same researcher on both occasions.

#### VQ11

The VQ11 is a brief, self-administered HRQoL questionnaire that was specifically designed to allow individual monitoring of COPD patients over a short-term period. The questionnaire’s preliminary versions were developed according to the standard stages of questionnaire validation [20,21], including evaluation of content validity, item clarity and construct validity [15]. An initial version of the questionnaire was drawn up by a panel of experts consisting of 20 COPD professionals and 15 patients with different degrees of COPD and different psychosocial levels. The clarity of each item was then tested on 20 patients with different degrees of COPD and different psychosocial levels. After making adjustments to the initial questionnaire, the committee produced an experimental questionnaire with 24 items, covering three theoretical components (functional, psychological and relational) and 11 sub-components. Each sub-component consisted of two or three items. This experimental questionnaire was tested on 166 COPD patients. Confirmatory factor analysis showed that the best model was a two-level hierarchical model with an initial level comprising 11 items (one per sub-component) distributed across three components (functional = 3 items; psychological = 4 items; social = 4 items) and a top level (lower score indicates better HRQoL) combining these three components. Cronbach’s alphas were calculated to test the internal consistency scales. The resulting values were 0.83 for the functional component, 0.69 for the psychological component, 0.57 for the social component and 0.83 for the total scale. Table 4 shows the French version of the VQ11 and a cross-cultural translation produced by three native speakers of English.

**Table 4:** Content and structure of the VQ11 questionnaire and its cross-cultural translation by three native speakers of English

	**English**	**French**
Information	The following sentences express feelings about the consequences of COPD. For each sentence, tick the intensity that best reflects your feeling at this moment (from “not at all” to “extremely”). There are no wrong answers. Each one is personal.	Les phrases suivantes expriment des sentiments sur les conséquences de la BPCO. Pour chacune, cochez l’intensité qui vous correspond le mieux maintenant (de « pas du tout » à « extrêmement »). Aucune réponse n’est juste. Elle est avant tout personnelle.
*Dyspnea*	*I suffer from breathlessness*	*Je souffre de mon essoufflement*
*Anxiety*	*I am worried about my respiratory condition*	*Je me fais du souci pour mon état respiratoire*
*Closeness*	*I feel my entourage (family, friends, etc.) misunderstands me*	*Je me sens incompris(e) par mon entourage*
*Mobility*	*My respiratory condition prevents me from moving about as easily as I would like*	*Mon état respiratoire m’empêche de me déplacer comme je le voudrais*
*Sleep*	*I feel sleepy during the day*	*Je suis somnolent(e) dans la journée*
*Life project*	*I feel unable to achieve my objectives*	*Je me sens incapable de réaliser mes projets*
*Fatigue*	*I quickly get tired when doing day-to-day activities*	*Je me fatigue rapidement dans les activités de la vie quotidienne*
*Physical confidence*	*Physically, I am dissatisfied with what I can do*	*Physiquement. je suis insatisfait(e) de ce que je peux faire*
*Social life*	*My respiratory disease disrupts my social life*	*Ma maladie respiratoire perturbe ma vie sociale*
*Depression*	*I feel sad*	*Je me sens triste*
*Emotional life*	*My respiratory condition restricts my emotional life*	*Mon état respiratoire limite ma vie affective*

#### Other questionnaires

The MOS-SF-36 [22,23], the SGRQ-C [24], the HADS [25], and the PSW of the French version [26] of the Physical Self-Perception Profile [27] were assessed.

#### MMRC scale

The degree of dyspnea was measured using the Modified Medical Research Council (MMRC) scale [28], which correlates well with other scales and health status scores [29].

### Analyses of respiratory function

The pulmonary function tests (PFTs) included simple screening spirometry, formal lung volume measurement, diffusing capacity for carbon monoxide, and arterial blood gases. We measured the volume-time curve and the flow-volume loop, as well as FVC and FEV_1_ in order to calculate FEV_1_/FVC indices [30].

### Exercise tolerance

The 6MWT test was performed twice with more than 30 minutes between tests in order to allow heart rate and dyspnea to return to their initial rest values [31]. A dyspnea score was measured on a 10-cm visual analog scale (VAS) before and immediately after the test.

### Statistical analyses

CFA was used to confirm the theoretical model found during the internal validation (Statistical Software Mplus 5.1). Fit assessment of the CFA models was based on multiple indicators [32-34], including the Chi-square statistic (χ^2^), comparative fit index (CFI), Tucker-Lewis Index (TLI), root mean square error of approximation (RMSEA), and 90% confidence interval (CI) of the RMSEA. Values greater than 0.90 for CFI and TLI are considered to indicate adequate model fit, although values approaching 0.95 are preferable. Values smaller than 0.08 or 0.06 for the RMSEA indicate acceptable and good model fit, respectively [33,34]. For the RMSEA 90% CI, values less than 0.05 for the lower bound (left side) and less than 0.08 for the upper bound (right side) or of 0 for the lower bound and less than 0.05 for the upper bound (right side) indicate acceptable and good model fit, respectively [35]. Factor loadings, squared multiple correlations, standard errors and t values were inspected for appropriate sign and/or magnitude.

Concurrent validity was assessed by analyzing Pearson correlation coefficients between the dimensions of the study questionnaire and those of another questionnaire measuring similar concepts. Spearman correlation coefficients were calculated between the VQ11 (total and component scores) and other independent variables. In order to confirm good concurrent validity but no redundancy, the new questionnaire had to show moderate correlation (0.40 to 0.70) with a well-established mea surement tool.

Reliability is the degree to which an instrument is free from random error. It is evaluated by measuring internal consistency reliability and reproducibility. Internal consistency reliability refers to the homogeneity of the items of the scale and was assessed using Cronbach’s alpha. Reproducibility establishes the stability of an instrument over time in a stable population and was tested using a Pearson correlation.

## Results

### Patient demographics

One hundred eighty-one participants completed the study baseline questionnaires and 49 participants completed the follow-up questionnaires. The mean ± SD and range of the physiological and patient-reported outcomes for the study population are summarized in Table 3.

### Factor validity

**Figure 1:**
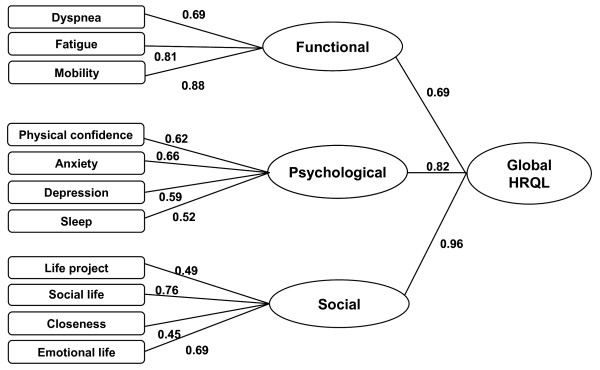
Hierarchical model and indices obtained for the VQ11 using confirmatory factor analysis (n = 181 COPD patients).

A CFA supported the validity of a two-level hierarchical model with a three-component initial level and a single top level (Figure 1). The weighted least squares mean- and variance-adjusted χ^2^ estimator analyzed all the items as categorical variables. Fit indices were acceptable (χ^2^ = 133.090; df = 24; CFI = 0.910, TLI = 0.955; RMSEA = 0.158). The fit for the one-factor model was as good as the fit for the three-factor model (χ^2^ = 135.573; df = 25; CFI = 0.909, TLI = 0.956; RMSEA = 0.156), but it was less acceptable with the difftest (difftest = 12.277; df: 3; p = 0.0065).

Reproducibility was assessed in terms of the correlations between the measures produced by the 49 COPD patients who were reassessed after a one-week period (42 patients under 10% of variation; 7 patients under 20% of variation). Correlation coefficients were 0.76 for the functional component (p < .01), 0.65 for the psychological component (p < .01), 0.73 for the social component (p < .01), and 0.88 for total VQ11 (p < .01).

### Reliability

Cronbach’s alphas were 0.80 for the functional component, 0.68 for the psychological component, 0.77 for the social component and 0.89 for the whole scale. Alphas computed using the Spearman-Brown formula were 0.91, 0.81 and 0.87, respectively.

The low correlations between VQ11 and FEV_1_ (functional component, -0.26; total, -0.19) suggest that there are no significant differences between groups classified by grade of severity and VQ11 score.

Table 5 presents the clinical characteristics and HRQoL measures of the 181 COPD participants according to quartiles of VQ11 score. Associations between increasing VQ11 quartiles and clinical characteristics were found for FEV1, pre-BD FEV1, FEV1/FVC and 6MWT distance (negative association), as well as for BODE score and dyspnea from both MMRC classification and the 6MWT-end measure (positive association). There were no significant associations between VQ11 quartiles and either age or BMI. HRQoL measures from SGRQ were all positively and significantly associated with VQ11 quartiles including SGRQ symptoms, activity, impact and total scores (p < 0.0001).

**Table 5:** Clinical characteristics and HRQoL measures by quartiles of VQ11 score

**VQ11 quartiles**	**Q1(≤20)**			**Q2 (20–28)**			**Q3 (28–34.5)**			**Q4 (>34.5)**			Scheffe^**^	***F***
	**(N = 45)**			**(N = 51)**			**(N = 37)**			**(N = 44)**				***P value***^$^
*Clinical characteristics*														
Age (yrs)	62.1	±	9.1*	60.9	±	*10.6*	61.2	±	*11.0*	61.6	±	*8.1*		0.94
BMI (kg/m^2^)	25.3	±	*5.0*	25.9	±	*4.7*	25.0	±	*5.4*	26.6	±	*6.3*		0.55
MMRC Dyspnea	0.36	±	*0.77*	1.23	±	*1.26*	1.8	±	*1.2*	2.4	±	*1.1*	Q1 < Q3; Q1 < Q2 < Q4	<0.0001
Pre-BD FEV_1_ (ml)	1676	±	*727*	1413	±	*586*	1176	±	*576*	1267	±	*590*	Q1 < Q3,Q4	0.002
FEV_1_ (% pred)	55.8	±	*18.7*	51.4	±	*21.6*	43.5	±	*19.2*	44.2	±	*20.5*	NS	0.013
FEV_1_/FVC (%)	53.1	±	*10.3*	47.5	±	*13.2*	44.4	±	*11.7*	45.5	±	*12.6*	Q1 < Q3,Q4	0.006
Dyspnea 6MWD end	5.2	±	*1.9*	6.1	±	*1.9*	5.9	±	*2.1*	6.7	±	*2.2*	Q1 < Q4	0.026
6MWT distance (m)	521	±	*85.0*	504	±	*119*	449	±	*111*	403	±	*132*	Q1,Q2 < Q4	<0.0001
BODE	1.8	±	*1.6*	3.1	±	*1.9*	4.2	±	*2.2*	5.2	±	*2.4*	Q1,Q2 < Q4; Q1 < Q2,Q3	<0.0001
*HRQoL Measures*														
SGRQ-C Symptoms	43.1	±	*15.3*	52.5	±	*17.1*	59.1	±	*16.8*	64.0	±	*20.0*	Q1 < Q3,Q4; Q2 < Q4	<0.0001
SGRQ-C Activity	38.9	±	*17.2*	51.8	±	*19.4*	60.7	±	*18.9*	71.5	±	*21.9*	Q1 < Q2 < Q4; Q1 < Q3	<0.0001
SGRQ-C Impact	16.3	±	*9.2*	31.6	±	*13.7*	42.3	±	*16.8*	48.9	±	*16.5*	Q1 < Q2 < Q3,Q4	<0.0001
SGRQ-C Total	28.9	±	*10.6*	43.3	±	*13.5*	53.4	±	*14.6*	61.4	±	*17.0*	Q1 < Q2 < Q3,Q4	<0.0001

*Mean ± *SD.*

**Significant at 0.05 level.

^$^Analysis of covariance for variables showing both normal distributions and homogeneous variance; Kruskal-Wallis test for variables showing normal distributions and/or homogeneous variance.

### Construct validity

The construct validity results are shown in Tables 6 and 7. The VQ11 showed good correlation with SGRQ total scores (0.71), SF-36 component scores (-0.61 for MCS and -0.61 for PCF), HADS total scores (0.61), and physical self-worth (-0.59). SGRQ total scores correlated most strongly with the functional component of the VQ11 (0.66), whereas MCS and HADS scores correlated most strongly with the psychological and social components of the VQ11 (-0.61 and 0.63; -0.60 and 0.61 respectively). Furthermore, we found significant correlations between VQ11 total scores and 6MWT end dyspnea scores, 6MWT distances (in meters and in percentage of the predicted distance) and total BODE scores (0.26, -0.37, 0.51 respectively). These correlations were particularly satisfactory for the functional component of the VQ11 (0.28, -0.42, 0.56 respectively). Correlations with FEV_1_, FEV_1_ as a percentage of the predicted value, FEV_1_/FVC and pack-years for smokers were low.

**Table 6:** Correlation between VQ11 and other variables of interest

	**Functional**	**Psychological**	**Social**	**Total**
Age	0.10	-0.11	-0.08	-0.04
BMI	0.13	0.05	0.05	0.08
Pack-years (smokers)	0.01	0.10	0.06	0.06
Pack-years (ex-smokers)	**0.29**	0.19	**0.20**	**0.25**
Pre-BD FEV_1_	**-0.28**	-0.14	**-0.16**	**-0.21**
FEV_1_ % pred	**-0.26**	-0.14	-0.13	**-0.19**
FEV_1_/FVC	**-0.26**	**-0.17**	**-0.16**	**-0.22**
MMRC Dyspnea Grade	**0.61**	**0.49**	**0.51**	**0.59**
Dyspnea 6MWD start	**0.17**	0.04	0.10	0.11
Dyspnea 6MWD end	**0.28**	**0.20**	**0.24**	**0.26**
Dyspnea 6MWD difference	**0.17**	**0.19**	**0.16**	**0.19**
6MWT distance (m)	**-0.42**	**-0.25**	**-0.34**	**-0.37**
6MWT distance (% pred)	**-0.41**	**-0.26**	**-0.35**	**-0.38**
BODE Index	**0.56**	**0.41**	**0.43**	**0.51**

In bold type, p < .05.

**Table 7:** Correlation between VQ11 and other questionnaire scores

	**Functional**	**Psychological**	**Social**	**Total**
*SGRQ-C*				
Symptoms	**0.43**	**0.41**	**0.40**	**0.46**
Activity	**0.57**	**0.48**	**0.54**	**0.59**
Impact	**0.63**	**0.60**	**0.64**	**0.69**
Total	**0.66**	**0.61**	**0.65**	**0.71**
*MOS SF-36*				
Physical functioning	**-0.64**	**-0.50**	**-0.53**	**-0.62**
Physical role	**-0.45**	**-0.38**	**-0.36**	**-0.43**
Emotional role	**-0.28**	**-0.42**	**-0.36**	**-0.39**
Energy/vitality	**-0.44**	**-0.56**	**-0.54**	**-0.58**
Mental health	**-0.21**	**-0.46**	**-0.41**	**-0.41**
Social functioning	**-0.40**	**-0.53**	**-0.63**	**-0.59**
Bodily pain	**-0.40**	**-0.38**	**-0.46**	**-0.46**
General health perceptions	**-0.42**	**-0.54**	**-0.51**	**-0.55**
Physical Component Scale	**-0.52**	**-0.57**	**-0.56**	**-0.61**
Mental Component Scale	**-0.42**	**-0.61**	**-0.60**	**-0.61**
*HADS*				
Anxiety	**0.21**	**0.49**	**0.46**	**0.44**
Depression	**0.47**	**0.59**	**0.58**	**0.62**
Total	**0.39**	**0.63**	**0.61**	**0.61**
*Physical Self-Inventory*				
Physical self-worth	**-0.47**	**-0.62**	**-0.50**	**-0.59**

In bold type, p < .05.

## Discussion

This study examined the validity and reliability of the VQ11, a short, self-administered questionnaire specifically designed for repeated assessment of patients with COPD and for use in routine care. The results show that the VQ11 provides a simple and reliable measure of overall COPD-related HRQoL and physical, psychological, and social components of HRQoL as expected by experts [17].

The hierarchical structure of a preliminary version of the VQ11 had previously been tested on a sample of 166 COPD patients [15]. Therefore, the initial aim of the present study was to use CFA to verify this factorial structure with a new sample of COPD patients. Our findings demonstrated that the higher-order factor model provided a satisfactory fit to the data and a better fit than the alternative models. These results confirm those from a previous study [15]. The model contains physical, psychological, and social components, in line with guidelines for HRQoL questionnaires [17,22,34]. Our analysis showed that these components can be examined separately.

The concurrent validity of the VQ11 was confirmed by the correlation between VQ11 scores (for the individual components and for the total score) and the SF-36 or the SGRQ-C. VQ11 total scores strongly correlated with scores for the physical and mental components of the SF-36, and with scores on the eight scales that make up these components. As expected, the highest correlation was for the physical functioning scale [36]. Also as expected, the highest correlations were between corresponding components of the VQ11 and the SF-36 (VQ11-functional scale and the SF-36 physical component and physical functioning scales; VQ11-psychological scale and SF-36 emotional role, energy/vitality, mental health, general health perceptions and mental component scales; VQ11-social scale and SF-36 social functioning scale). By contrast, the correlation between the component and total scores on the VQ11 and the SF-36 bodily pain scale was not particularly strong. However, because the SF-36 was not designed to measure sleep disturbances and respiratory complaints, this relatively weak correlation does not affect the concurrent validity of the VQ11. As expected, VQ11 total scores correlated well with SGRQ-C scores and three specific domains.

As expected also [37], VQ11 total scores correlated well with HADS depression and total scores. The results also supported the weak correlations between airway obstruction and HRQoL [7,38,39].

The three components of the VQ11 are disease-specific domains of HRQoL for COPD patients. VQ11 functional scores correlated with dyspnea (MMRC grade and 6MWT distance), BODE index, exercise tolerance (6MWT distance), the activity and impact scores of the SGRQ-C, and the physical functioning and physical components of the SF-36. The items of the VQ11 reflect the main symptoms perceived by patients with COPD, dyspnea [28], physical limitation [24] and fatigue [37,38,40].

Significant correlations were found between the psychological component of the VQ11 and the HADS anxiety and depression scales, the SGRQ-C impact scale, the emotional role and mental components of the SF36, and physical self-worth (defined as physical self-esteem). This emphasizes that disease-associated anxiety and depression are important HRQoL factors for COPD patients. The degree of anxiety felt by COPD patients has been shown to be related to their degree of pulmonary dysfunction [41].

The social component of VQ11 correlated with the social functioning and mental components of the SF36. Social relationships are affected by chronic respiratory disease, especially in patients with severe respiratory insufficiency who often depend on close social relationships to manage daily activities [42]. Patients with COPD experience losses in several areas of their lives, and they may feel useless, experience reduced sexual activity, depend on others for their personal care and lose interest in future projects.

The total, functional, psychological, and social components of the VQ11 showed good reliability over a period of one week for patients without clinical change. The correlation coefficient for the psychological component was the lowest. This result can be explained by intra-individual variability in the perception of disease and health status. A recent study noted higher day-to-day instability in self-esteem, which is a major correlate factor of HRQoL for COPD patients compared with healthy adults [41]. Consequently, it would be advisable for clinicians to ask patients to complete the VQ11 every three to six months, in order to assess the stability of patients’ perceptions of their illness, as this perception may be a sign of vulnerability and of the likelihood they will not fully adhere to their treatment.

There were limitations to the study. The sample was relatively homogenous, with all subjects having moderate to severe COPD, the majority being ex-smokers with a significant smoking history. The concept of quality of life and its implications on daily life are different for men and women [21]. We could not validate the new questionnaire separately for men and women due to the small sample size. The VQ11 also needs to be studied in other ethnic populations and cultures for cross-cultural validity. In addition, the responsiveness of the VQ11 to interventions and comparisons with other quality of life questionnaires is also required. Last, using a new patient-reported outcomes questionnaire requires the determination of the minimal clinically important difference [43].

In practice, there are four general benefits to using the VQ11: (1) It helps clinicians quickly detect the worsening of HRQoL in COPD patients. This deterioration can then be explained by the acknowledgment of COPD, poor disease self-management (routine or acute situation), the presence of comorbidities (depression, sleep trouble, metabolic syndrome…) and/or weak support from family and friends. Possible consequences include an increase in the exacerbation risk and aggravation of COPD, the development of health-risk behavior or of a new disease, and/or the deterioration of communication with caregivers, family or friends. (2) The three components of the VQ11 allow caregivers to assess and correct a number of situations: based on a high score on the functional component, informed decisions can be made on therapy, modification of current treatment, new assessment, physiotherapy or comprehensive rehabilitation; with a high score on the psychological component, those decisions can be made regarding new assessment, psychological support, education, or comprehensive rehabilitation; finally, with a high score on the social component, decisions can be made concerning social support, psychological support, education, membership in a health network or patients association. (3) Based on the anticipated validation of a Minimal Clinically Important Difference, the VQ11 can also help monitor the efficacy of an individual’s therapeutic decision. (4) Lastly, the VQ11 provides clinicians with meaningful cues to examine a COPD life consequence more specifically when an answer to an item is more than three. Moreover, the back of the form includes an educational message with a space for drawing and commenting in which individual messages can be exchanged between patients and caregivers, family or friends.

## Conclusions

This study showed the validity and reliability of the VQ11, a short, self-administered questionnaire specifically designed for repeated assessment of patients with COPD and for use in routine care. The VQ11 provides clinicians and patients with a simple and reliable measure of overall COPD-related HRQoL. The VQ11 facilitates discussions about the overall consequences of COPD arising from the illness’s physical symptoms and psychological perceptions, observance behaviors, health behaviors, life projects with COPD, and social support. Additional information is needed to provide responsiveness to change at the individual patient level, an essential feature for its use in clinical practice.

## Abbreviations

BMI: Body mass index; COPD: Chronic obstructive pulmonary disease; CRDQ: Chronic respiratory disease questionnaire; CFI: Comparative fit index; CAT: COPD assessment test; CI: Confidence interval; CFA: Confirmatory factor analysis; CCQ: COPD clinical questionnaire; FEV1: Forced expiratory volume in 1 s; FVC: Forced vital capacity; HRQoL: Health-related quality of life; HADS: Hospitalization anxiety depression scale; MMRC: Modified medical research council; PSW: Physical self-worth; PFTs: Pulmonary function tests; RMSEA: Root mean square error of approximation; SF-CRQ: Short form chronic respiratory disease questionnaire; 6MWT: Six-minute walk test; SGRQ: St George’s respiratory questionnaire; TLI: Tucker-Lewis index; VAS: Visual analog scale; VSRQ: Visual simplified respiratory questionnaire.

## Competing interests

The authors declare that they have no competing interests.

## Authors’ contributions

GN conceived the study, participated in its design, performed the statistical analysis, and coordinated the drafting of the manuscript. FS and CP participated in the design of the study and participated in drafting the manuscript. All authors read and approved the final manuscript.

## Institutions at which the work was carried out

Service Central de Physiologie Clinique, Unité d’Exploration Respiratoire. CHRU Arnaud de Villeneuve, Montpellier

CHRU Béclère, Clamart

CHRU St-Antoine, Paris

Cabinet de pneumologie, Bagneux

Clinique du Souffle La Solane, Osséja

Clinique du Souffle La Vallonie, Lodève

Réseau AIR + R, Montpellier
